# Risk factors for unresectable pancreatic cancer following high‐intensity focused ultrasound treatment

**DOI:** 10.1002/cam4.6568

**Published:** 2023-10-04

**Authors:** Yu Yang, Xian‐quan Shi, Guang Chen, Lin‐xue Qian

**Affiliations:** ^1^ Department of Ultrasound, Beijing Friendship Hospital Capital Medical University Beijing China; ^2^ Department of Interventional Radiology, Beijing Friendship Hospital Capital Medical University Beijing China

**Keywords:** high‐intensity focused ultrasound, lymphocyte subsets, overall survival, pain, unresectable pancreatic cancer

## Abstract

**Purpose:**

Pancreatic cancer is one of the most aggressive malignant tumors with poor prognosis. High‐intensity focused ultrasound (HIFU) is an effective and safe treatment option for advanced pancreatic cancer, however, the survival time of patients after the treatment was different. So, the purpose of this study was to evaluate the relationship between the high‐risk characteristics and prognosis of unresectable pancreatic cancer after HIFU treatment.

**Patients and Methods:**

This prospective study included 30 patients with unresectable pancreatic cancer who received HIFU at Beijing Friendship Hospital. Data on patients' tumor size, pain scores, peripheral blood lymphocyte subsets, CA19‐9 and contrast enhanced ultrasound (CEUS) features were collected to assess the relationship with overall survival (OS) after HIFU.

**Results:**

The median OS from the start of HIFU treatment was 159 days, 95% confidence interval (95% CI): 108–210. The levels of pain were determined by visual analogue scale (VAS) score, and the quartile of the score decreased from 6 (2, 7) to 4 (2, 5) immediately after one session of the treatment (*p* = 0.001). The diagnostic model showed that high post VAS score and decreasing of peripheral CD4^+^ T cells were significantly correlated with poor prognosis (*p* < 0.05), and showed good discrimination ability (AUC = 0.848, 95% CI = 0.709–0.987).

**Conclusion:**

HIFU can effectively relieve pain in patients with unresectable pancreatic cancer. Post treatment VAS and change of peripheral CD4^+^ T cells are independent risk factors affecting the prognosis in patients with unresectable pancreatic cancer after HIFU treatment.

## 1 INTRODUCTION

Pancreatic cancer is one of the most aggressive malignancies with a poor prognosis and a 5‐year survival rate of only 8%.^1^ According to the 2018 Global Cancer Statistics, pancreatic cancer is the seventh leading cause of cancer‐related death worldwide, and surgical resection is the only means of cure.^2^ Owing to hidden symptoms, only 15%–20% of patients have resectable lesions at the initial diagnosis. Advanced pancreatic cancer usually invades adjacent important structures, especially the celiac trunk and superior mesenteric artery, and the median overall survival (OS) of untreated patients with locally advanced pancreatic cancer is 8–12 months.^3^ Existing research shows that high‐intensity focused ultrasound (HIFU) is an effective and safe treatment for unresectable pancreatic cancer, because it can effectively relieve patients' pain, reduce serum carbohydrate antigen (CA) 19‐9 level, improve quality of life, and reduce tumor volume.^4^ However, it is still a problem to be solved that which characteristics of patients can benefit more from this treatment.

An important reason for the poor prognosis of patients with pancreatic cancer is the dense fibrotic stroma, makes up to 90% of the tumor mass, which increases interstitial pressure in the tumor, collapses the vasculature, and reduces the penetration of drugs and effector immune cells.^5^ Pancreatic cancer is often considered immunologically “cold”, but the progression of pancreatic cancer has a close correlation with the immune system.^6^ In anticancer immunity, the complex interaction between immune cells such as NK, CD4^+^ T, and CD8^+^ T cells can significantly affect the survival rate, which has been reported in tumor‐infiltrating lymphocytes in pancreatic cancer.^7^ Encouragingly, locoregional therapies have been shown to induce changes in the structure, composition and properties of the pancreatic cancer tumor microenvironment, which may help in the treatment of the disease.^8^ HIFU uses a focused ultrasound beam to create a thermal effect at the lesion, which can cause rapid coagulative necrosis with limited inflammatory response and minimal damage to outside the focal area. At the same time, the hyperthermia produced by the action of HIFU increases blood flow and vascular permeability, disrupt the stromal architecture, thus improving anti‐tumor effects.^8^ HIFU also uses the cavitation and mechanical effect of ultrasound to increase vascular permeability, promote drugs penetration,^9^ induce subcellular fragmentation, and trigger activation of cytotoxic T cells in other types of tumors.^10^, ^11^, ^12^

However, it is very difficult to obtain the tumor‐infiltrating lymphocytes in pancreatic cancer in clinical work. In contrast, the information of patient's peripheral blood lymphocyte subsets is easy to get in clinical work. Recent studies have shown that peripheral blood lymphocytes play an important role in predicting OS and evaluating the therapeutic effect.^13^ Cancer‐related pain exists in 60%–90% of patients with pancreatic cancer at the time of diagnosis, which seriously reduces the patients' quality of life and negatively affects their survival.^14^ Previous research has shown that the pain symptoms of patients with unresectable pancreatic cancer may be relieved immediately after the treatment with HIFU.^15^ In addition, previous studies have also confirmed that the tumor size, location, and patient serum markers of pancreatic cancer have a important clinical significance in predicting prognosis.^16^, ^17^

Therefore, this study aimed at evaluate the relationship between these high‐risk characteristics and prognosis of unresectable pancreatic cancer after HIFU treatment, in order to provide help for HIFU treatment of unresectable pancreatic cancer in the future.

## 2 MATERIALS AND METHODS

### 2.1 Patients

This is an exploratory study, and prospectively enrolled patients with unresectable pancreatic cancer in our hospital from January 2020 to January 2022. All patients received regular pancreatic arterial infusion chemotherapy in the interventional radiology department of our hospital except for HIFU treatment. The inclusion criteria were as follows: (1) age of 18–75 years; (2) with normal coagulation function; (3) diagnosis had been confirmed by puncture biopsy or intraoperative exploration; (4) no other treatments are used on the day of HIFU treatment, in other words, HIFU and arterial infusion chemotherapy cannot be given on the same day to avoid interfering with the study results. The following patients were excluded from this study: (1) tumor could not be clearly displayed by the built‐in ultrasonic machine of the HIFU instrument; (2) with a poor general condition; (3) who were unable to cooperate or complete treatment, had heart, liver, lung, kidney, other organ failure; (4) had immune deficiencies. The primary endpoint of this study was OS, defined as the time from the first day of HIFU treatment until death from any cause. The study protocol was approved by the Human Ethics Review Committee of the Beijing Friendship Hospital (2019‐P2‐226‐02) and has been registered in the Chinese Clinical Trial Registry (ChiCTR2000029620). The study design and implementation were in compliance with The Code of Ethics of the World Medical Association (Declaration of Helsinki). Written informed consent was obtained from all the patients before enrollment.

### 2.2 Research protocol

**FIGURE 1:**
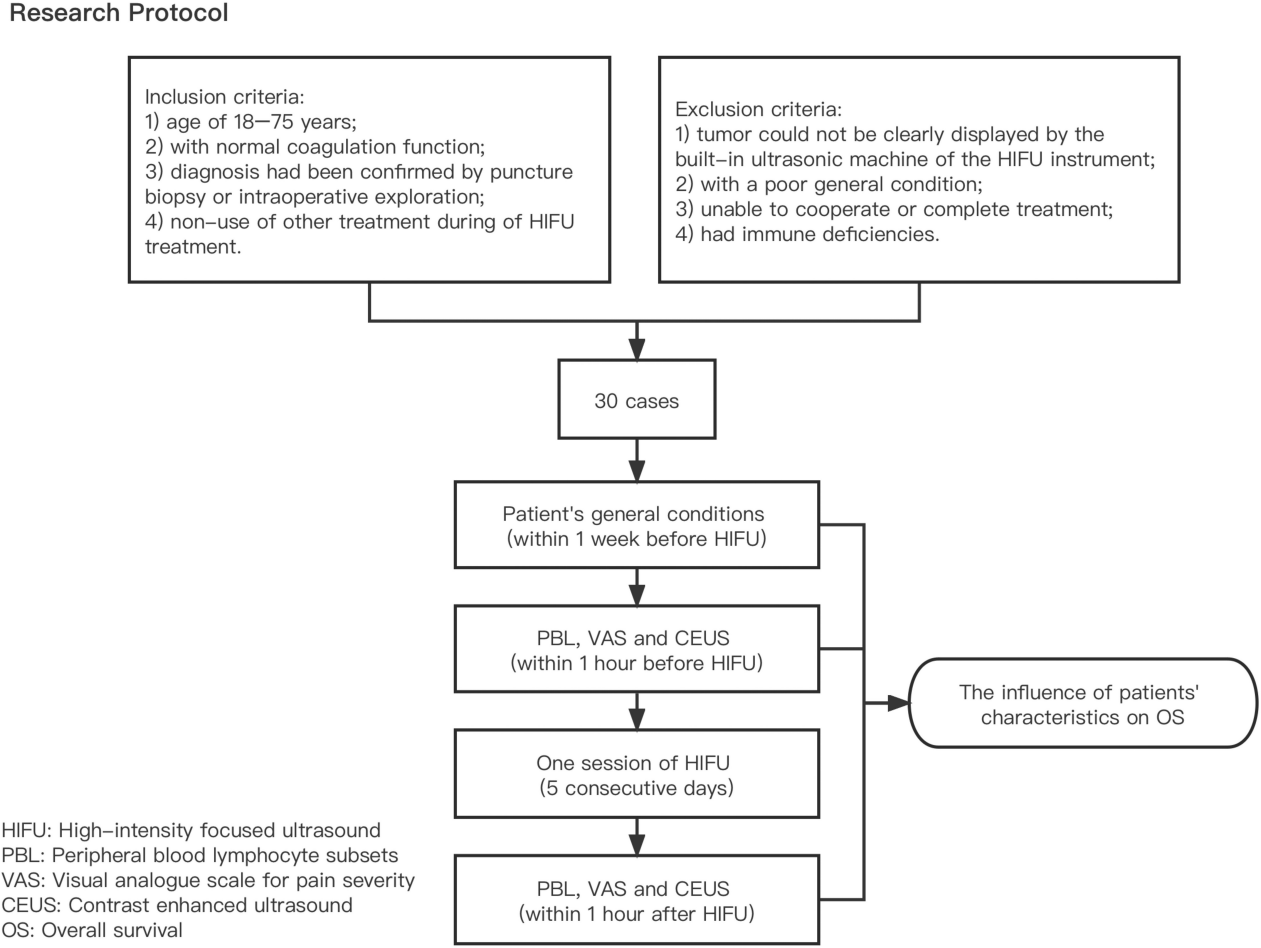
Research protocol.

Peripheral blood lymphocyte subsets, pain's visual analogue scale (VAS) and contrast enhanced ultrasound (CEUS) features were collected within 1 h before a session of HIFU and after treatment. Each session of HIFU treatment was conducted for 5 consecutive days. Patients' general information, such as sex, age, stage, serum CA19‐9, body mass index (BMI), tumor size and existence of metastasis were obtained within 1 week before treatment. And then, the correlation between these risk factors and OS were analyzed. The flow chart of this study is as follows (Figure 1).

### 2.3 Procedure of HIFU

**FIGURE 2:**
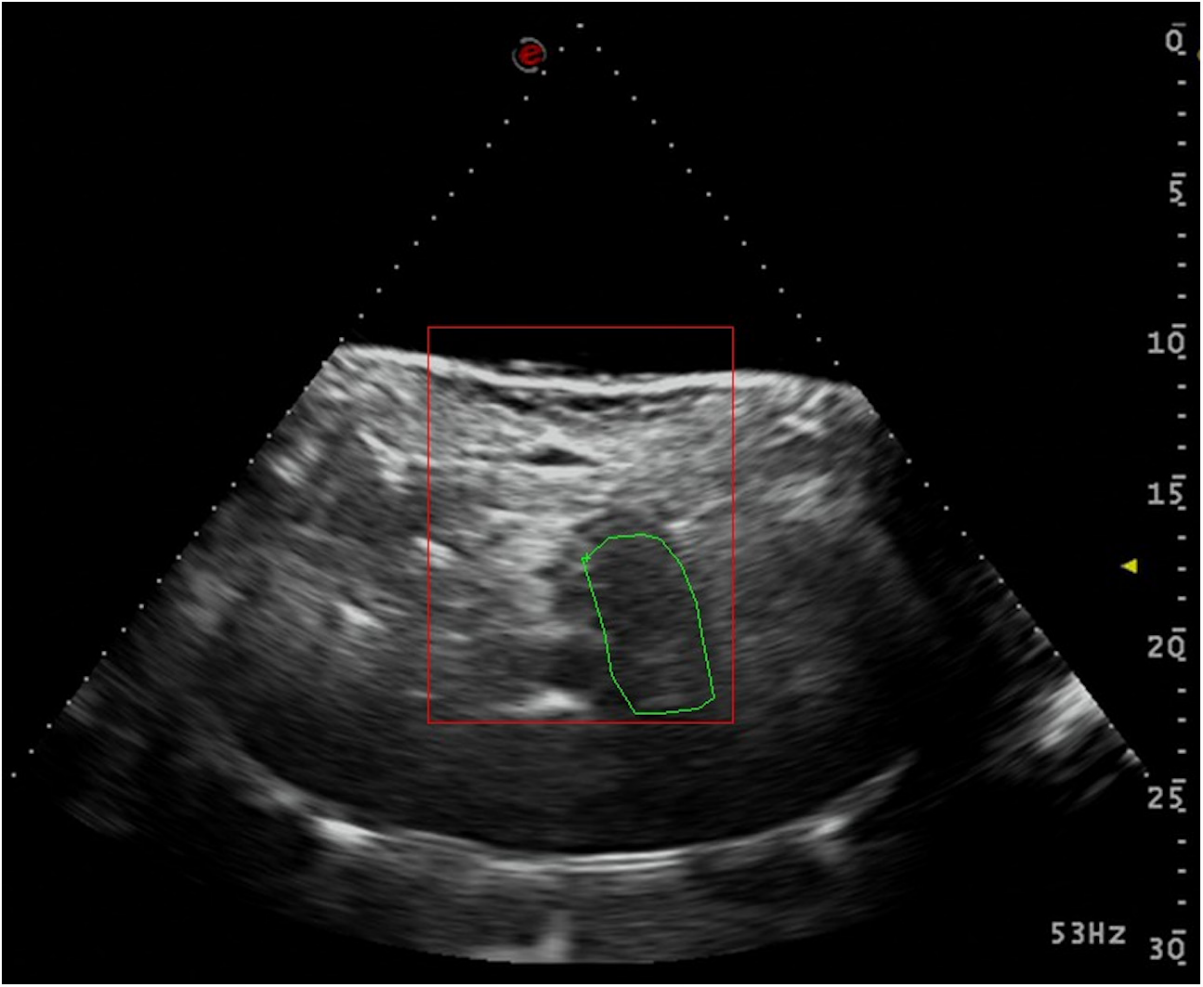
The guiding image during high‐intensity focused ultrasound (HIFU) treatment. The doctor delineated the target treatment area (in the green circle) on the software based on the ultrasound image.

The procedure was performed on an ultrasound guided HIFU system. Pre‐treatment planning and real‐time image guidance was performed by Esaote MyLab class C diagnostic ultrasound system (EsaoteTM). Treatment was delivered with the HIFUINT‐9000 system (Shanghai A&S Sci‐Tec Co., Ltd.), which consists of six self‐focusing transducers with a geometric radius of 200 cm and a treatment depth range of 0–140 cm, operating at a frequency of 1 MHz. The treatment and interval times were set at 200 and 400 ms respectively. The size of each treatment unit is 3 × 3 × 8 mm. During treatment, the patient lies supine on the treatment bed. Then the doctor controlled the treatment bed to the designated position, and when the tumor was clearly shown, treatment area can be drawn on the software. To ensure the safety, the treatment zone should be at least 5 mm away from the tumor edge (Figure 2).

### 2.4 Pain assessment

VAS was used to evaluate patients' pain. It consists of 11 points ranging from 0 to 10, where a score of 0 indicates no pain and scores 1–10 indicate pain aggravation. All patients were evaluated before and after one session of HIFU treatment. According to previous studies, when the patient's pain score is 1–3, it is defined as mild pain, 4–6 as moderate pain, and 7–10 as severe pain.^18^

### 2.5 Peripheral blood lymphocyte subsets

Within 1 h before a session of HIFU and after treatment, 4 mL of venous blood was reserved from each patient. Monoclonal antibodies were labeled with fluorescent dyes to determine various lymphocyte envelope differentiation antigens. The relative counts (percentage) of T cells (CD3^+^), B cells (CD3^−^ CD19^+^), NK cells (CD3^−^ CD16^+^ CD56^+^), CD4^+^ T cells (CD3^+^ CD4^+^) and CD8^+^ T (CD3^+^ CD8^+^) cells were determined. In our hospital, their normal value ranges are as follows: T cells (58.60%–83.10%), B cells (3.50%–15.40%), NK cells (6.90%–37.90%), CD4^+^ T cells (27.10%–49.80%), CD8^+^ T cells (19.40%–41.10%), and CD4/CD8 ratio (0.70–2.00).

### 2.6 CEUS

CEUS was performed before the patient was treated with HIFU and immediately after completing a session of treatment. The Hitachi ARIETTA 70 ultrasound diagnostic instrument was used for CEUS examination. The probe used in this study has a frequency range of 5–1 MHz and a mechanical index of 0.09. Sulfur hexafluoride microbubbles (SonoVue®, Bracco) was used as ultrasound contrast agent. 2.4 mL of SonoVue suspension was injected through the superficial vein of the elbow, and then the intravenous indwelling tube was quickly rinsed with 10 mL of sterile saline. The time intensity curve (TIC) was generated by the built‐in software of the Hitachi ARIETTA 70 ultrasound diagnostic instrument. Since the normal pancreatic tissue in this group of patients often atrophied, large blood vessels at the same level as the tumor were selected as references (such as the abdominal aorta, celiac trunk, etc.), and the relative rise intensity (rRI) was calculated, that is, intratumoral rise intensity (RI)/large blood vessel RI.

### 2.7 Others

Patients' general information, such as sex, age, body mass index (BMI), tumor size, tumor location, existence of metastasis and CA19‐9 were obtained. BMI was calculated using the following formula: weight (kg)/height (m^2^). The tumor size was defined as the maximum diameter of tumor. Based on the results of previous studies, patients were grouped according to their age <60 years or ≥60 years, tumor size <4 cm or ≥4 cm,^19^ tumor located in head/uncinate or body/tail, CA19‐9 <1000 or ≥1000 U/mL.^17^ OS was measured from the date of HIFU treatment until death.

### 2.8 Statistical analysis

SPSS 26.0 (IBM Corp.) and GraphPad Prism 9.0 (GraphPad, Inc.) were used for statistical analysis. An independent sample *t*‐test was used to compare normally distributed variables, and the Kruskal–Wallis H‐test was used for non‐normally distributed data. The median OS and potential risk factors were evaluated by the Kaplan–Meier method (univariate analysis), variables with a *p*‐value of <0.1 were analyzed using the multivariable Cox model. The receiver operating characteristic curve (ROC) was used to assess the discriminatory ability of the diagnostic model. *p* < 0.05 was considered statistically significant.

## 3 RESULTS

### 3.1 Baseline characteristics

A total of 30 patients were enrolled in this study. The baseline characteristics of patients are summarized in Table 1.

**TABLE 1:** Baseline characteristics of the patients.

Characteristics	All = 30
Age, years	62.73 ± 8.79 (42–75)
Sex, *n* (%)	
Women	14 (46.67%)
Men	16 (53.33%)
BMI, kg/m^2^	21.82 ± 2.45 (17.40–28.06)
Tumor location, *n* (%)	
Head/uncinate	20 (66.67%)
Body/tail	10 (33.33%)
Tumor size, cm	4.6 ± 1.7 (2.4–8.7)
CA19‐9, U/mL	339.95 (38.75, 1505.73)
Sites of metastatic, *n* (%)	
Liver	24 (80.00%)
Lymph node	14 (46.67%)
Lung	3 (10.00%)

Abbreviation: BMI, Body mass index.

### 3.2 VAS

**FIGURE 3:**
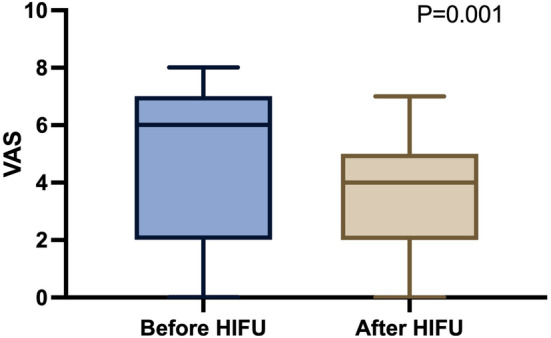
Difference in VAS before and after high‐intensity focused ultrasound (HIFU) treatment. VAS decreased significantly after treatment (*p* = 0.001).

One patient had no pain symptoms before and after HIFU treatment. Therefore, in the statistical analysis of pain changes, 29 patients with pain symptoms before treatment were counted. The quartile of VAS before HIFU treatment was 6 (2, 7) and decreased to 4 (2, 5) immediately after one session of the treatment; the difference was significant (*p* = 0.001, Figure 3).

### 3.3 Peripheral blood lymphocyte subsets

**FIGURE 4:**
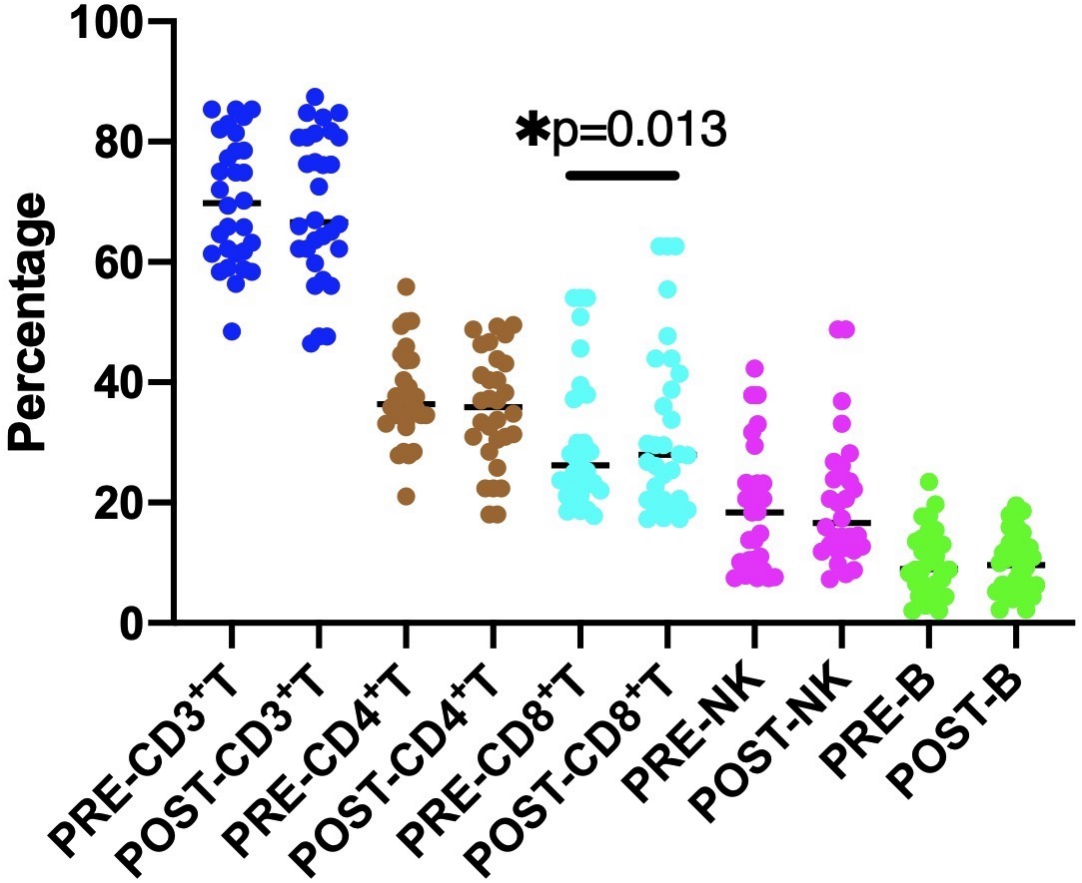
Peripheral blood lymphatic subsets before and after high‐intensity focused ultrasound (HIFU) treatment. CD8^+^ T cells increased after HIFU treatment (*p* = 0.013), and there were no significant differences in other parameters.

The results of peripheral blood lymphatic subsets before and after HIFU treatment are shown in Table 2 and Figure 4. The average percentage of CD8^+^ T cells increased from 30.30 ± 11.46 to 32.33 ± 14.26 after HIFU treatment (*p* = 0.013). There were no significant differences in other parameters.

**TABLE 2:** Peripheral blood lymphatic subsets before and after HIFU.

	Before HIFU	After HIFU	Decreased /increased	*p*‐value
CD3^+^ T cells (%)	70.11 ± 10.47	69.11 ± 12.09	16/14	0.355
CD4^+^ T cells (%)	37.65 ± 7.73	35.43 ± 9.38	19/11	0.085
CD8^+^ T cells (%)	30.30 ± 11.46	32.33 ± 14.26	13/17	0.013*
CD4^+^/CD8^+^	1.41 ± 0.60	1.35 ± 0.69	19/11	0.296
NK cells (%)	18.88 ± 10.19	20.10 ± 10.75	11/19	0.301
B cells (%)	10.13 ± 5.41	9.70 ± 4.99	18/12	0.412

Abbreviations: HIFU, high‐intensity focused ultrasound; NK, natural killer.

* *p* < 0.05.

### 3.4 CEUS

Compared with pretreatment, 14 patients demonstrated a reduction of rRI after a session of HIFU treatment, and the other 16 patients did not.

### 3.5 OS

The OS, which was calculated as the period from the start of HIFU, of all patients ranged from 33 to 318 days (median OS, 159 days), with a 95% confidence interval (CI) of 108–210 days.

### 3.6 Univariate analysis of high‐risk factors affecting OS

#### 3.6.1 Prognostic influence of the VAS score

**FIGURE 5:**
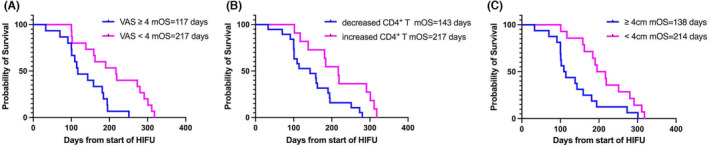
Kaplan–Meier survival curve. (A) Patients with visual analogue scale (VAS) score ≥4 (*p* = 0.004), (B) decreased CD4^+^ T cells (*p* = 0.012), and (C) tumor size ≥4 cm (*p* = 0.010) were significantly associated with shorter overall survival (OS).

All the patients were divided into two groups according to the pain symptoms before and immediately after treatment: the mild pain group (VAS score <4) and moderate‐to‐severe pain group (VAS score ≥4). After HIFU treatment, the median OS estimates of the mild pain group and moderate‐to‐severe pain group were 217 days (95% CI: 170–252) and 117 days (95% CI: 107–165), respectively. Survival curves were plotted by the Kaplan–Meier method, and the curves of patients in the moderate‐to‐severe pain were worse than those in the mild pain group (log‐rank test, *p* = 0.004) (Figure 5A). However, the OS distribution of different VAS groups before treatment was not statistically significant (Table 3).

**TABLE 3:** Univariate analysis of factors associated with OS.

Variable	Median survival (days)	Number of cases	Log‐rank test, *p*
Age, year			
<60	165	13	0.489
≥60	180	17	
Gender			
Women	178	14	0.425
Men	170	16	
Tumor size, cm			
<4	214	14	0.010*
≥4	138	16	
Tumor location			
Head/uncinate	171	20	0.517
Body/tail	178	10	
CA19‐9, U/mL			
<500	169	16	0.377
≥500	179	14	
VAS before HIFU			
<4	192	11	0.594
≥4	163	19	
VAS after HIFU			
<4	211	15	0.004*
≥4	136	15	
CD3^+^ T cells			
Decreased	149	16	0.123
Increased	201	14	
CD4^+^ T cells			
Decreased	149	19	0.012*
Increased	216	11	
CD8^+^ T cells			
Decreased	180	13	0.553
Increased	169	17	
CD4/CD8			
Decreased	164	19	0.317
Increased	190	11	
NK cells			
Decreased	200	11	0.529
Increased	158	19	
B cells			
Decreased	165	18	0.691
Increased	186	12	
rRI			
Decreased	157	14	0.365
Increased	188	16	

Abbreviations: HIFU, high‐intensity focused ultrasound; NK, natural killer; OS, overall survival; rRI, relative rise intensity of contrast enhanced ultrasound; VAS, visual analogue scale.

* *p* < 0.05.

#### 3.6.2 Prognostic influence of peripheral blood lymphocyte subsets

The patients were grouped depending on the increased or decreased of peripheral blood lymphocyte subset after HIFU treatment. Kaplan–Meier analysis showed that the patients with decreased CD4^+^ T cells had shorter OS (log‐rank test, *p* = 0.012) (Figure 5B). There was no significant difference in OS between the rising and falling groups of other types of cells (Table 3).

#### 3.6.3 Prognostic influence of tumor size

In this study, 14 patients with tumor size <4 cm and 16 patients with tumor size ≥4 cm. The estimated median OS was 214 days (95% CI: 178–250) in the group with tumor size<4 cm, and 138 days (95% CI: 103–173) in the group with tumor size ≥4 cm. The Kaplan–Meier method showed that the survival curve of patients with tumor size ≥4 cm was worse than that of patients with tumor size <4 cm (log rank test, *p* = 0.010) (Figure 5C).

#### 3.6.4 Others

There is no significant difference in OS distribution among different groups of age (*p* = 0.489), gender (*p* = 0.425), tumor location (*p* = 0.517), CA19‐9 (*p* = 0.350), and rRI (*p* = 0.365) (Table 3).

### 3.7 Multivariate survival analysis

Thus, VAS score after treatment, changes of CD4^+^ T cell and tumor size were analyzed by Cox model. The results showed that VAS score after treatment (HR = 1.394; 95% CI = 1.128–1.724; *p* = 0.002) and change of CD4^+^ T cell after HIFU (HR = 0.886; 95% CI = 0.816–0.961; *p* = 0.004) were independent predictors of OS (Table 4).

**TABLE 4:** Multivariate analysis of factors associated with OS.

Variable	Coefficient	HR	95% CI	*p*‐value
VAS after HIFU	0.332	1.394	1.128–1.724	0.002*
Change of CD4^+^ T cell	−0.121	0.886	0.816–0.961	0.004*

Abbreviations: CI, confidence interval; HIFU, high‐intensity focused ultrasound; HR, hazard ratio; OS, overall survival; VAS, visual analogue scale.

* *p* < 0.05.

**FIGURE 6:**
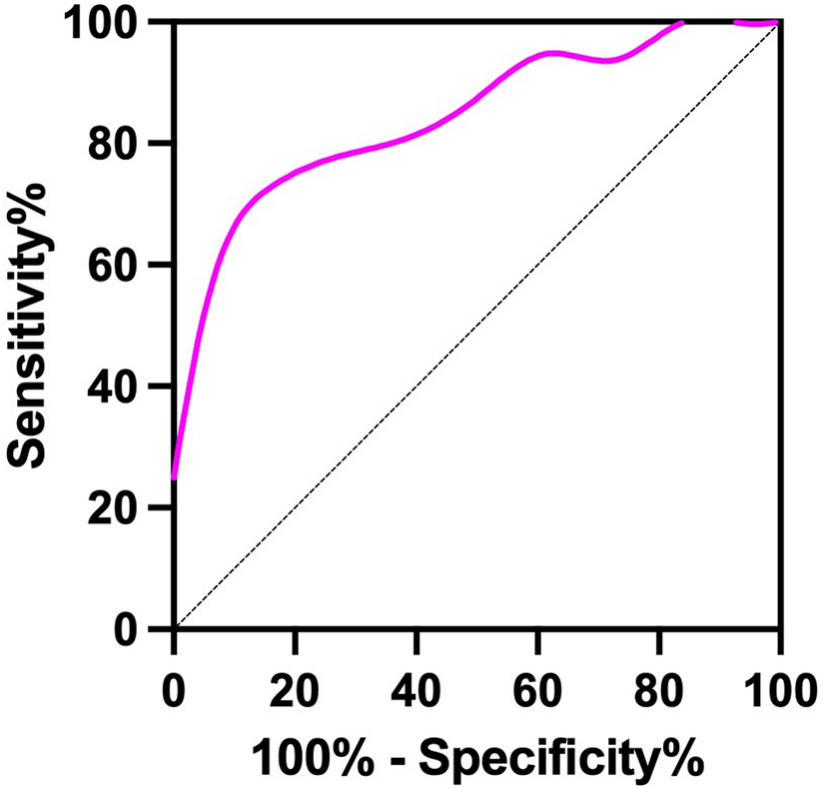
Receiver operating characteristic (ROC) curves to assess the discrimination ability of diagnostic model on 6‐month survival rate, and it showed good discrimination ability (AUC = 0.848, 95% CI = 0.709–0.987).

Since this study was aimed at patients with advanced pancreatic cancer and the median OS was 159 days. We analyzed the discrimination ability of diagnostic model on 6‐month survival rate by ROC curves, and it showed good discrimination ability (AUC = 0.848, 95% CI = 0.709–0.987) (Figure 6).

## 4 DISCUSSION

HIFU is one of the means to treat advanced pancreatic cancer. More and more studies have confirmed that it can alleviate the pain of patients, improve the quality of life of patients, and may extend the survival time of patients.^20^, ^21^ Our results also confirmed the effect of HIFU on pain relief in patients. In this study, we monitored the changes in VAS scores and peripheral blood lymphocyte subsets immediately after one session of HIFU treatment without any other intervening treatment. We also found that patients with high VAS score and decreased CD4^+^ T cells in peripheral blood after HIFU treatment had a poorer OS. Thus, the changes in the above indicators can objectively reflect the patient's response to HIFU.

The role of HIFU in reducing pain levels in patients with advanced pancreatic cancer has been confirmed by many studies.^22^ To explain the mechanism of HIFU‐mediated pain reduction in cancer patients, scholars have proposed the following hypotheses: (1) ablation‐induced fibrosis and tumor mass reduction reduce the pressure on nearby nerves to alleviate pain. (2) Heating of the tissue damages the nerve fibers conducting pain, reducing their density and leading to local denervation. (3) The sound pressure caused by the cavitation effect can temporarily change the cell morphology, lead to dysfunction of membrane proteins and stretch‐activated ion channels, cause imbalance of cation influx, and thereby change the membrane potential. HIFU can change the excitability of neurons and the transmission of the action potentials through these changes, thereby reducing the pain symptoms of patients.^23^

In our study, we evaluated the pain symptoms of patients before and immediately after treatment. Therefore, the reduction in peripheral nerve pressure caused by shrinkage of the pancreatic tumor had little effect on the reduction of pain symptoms of patients immediately after treatment and may even have the opposite effect due to the swelling of the treatment area. Therefore, we hypothesized that changes in the membrane proteins and ion channels induced by thermal injury, as well as damage to nerves by cavitation and mechanical effects, may be the reasons for the immediate relief of pain in the patients of this study. However, this hypothesis remains to be further verified by relevant experiments.

Tumor pain seriously affects the quality of life and OS of patients.^24^ For many cancer patients, pain is the most terrible symptom of the disease, especially for patients with advanced pancreatic cancer.^25^ Many studies in the literature have pointed out the influence of quality of life on survival rate. A large meta‐analysis of 30 trials for multiple cancer types found that pain was an important predictor of survival in multivariate analysis.^26^ The results reported by Ji et al. showed that the OS of patients with pain scores <4 was significantly better, which is consistent with our results.^4^ The potential mechanisms by which pain may promote cancer progression and shorten survival may include: (1) impaired host immune response induced by pain allowing easier growth and proliferation of malignant,^24^ (2) reduced ability of patients to receive intensive anti‐cancer treatment, and damaged pain‐mediated performance status, quality of life and nutritional intake,^27^ and/or (3). increased activation of multi‐opioid receptors through pain‐induced stimulation of endogenous opioids (such as endorphins), or increased consumption of opioid analgesics.^28^ Thus, the degree of pain will affect the OS of patients.

Tumor progression is a complex process involving tumor host immune interaction, and the host immune state profoundly affects tumor development, and vice versa.^29^ A successful antitumor immune response requires the coordination of a variety of cells that constitute the tumor microenvironment, including macrophages, DC, T cells, and B cells. Peripheral immune cells can reflect the immunity of tumor patients, and some studies have shown that the proportions of peripheral immune cells have an important influence on the prognosis of patients with different types of cancer.^13^, ^30^, ^31^ CD4^+^ T cells play an important role in antitumor immunity by regulating antigen‐specific immune response, thereby coordinating the complex antitumor immune process of inhibiting tumor progression and development.^32^, ^33^ For example, CD4^+^ helper T cells are responsible for providing regulatory cytokines required to activate CD8^+^ T cell soluble T lymphocytes, which are considered to be important effector cells for killing tumor cells.^34^ This approach can also enhance humoral immunity by activating B cells, thereby stimulating the production of antibodies.^35^ Similarly, CD4^+^ T cells appear to have significant plasticity, allowing subsets to switch to each other and expand their functional impact.^36^ Considering the role of CD4^+^ T cells in coordinating the response of each cell type, the potential influence of their deficiency is tremendous. Moreover, previous studies have shown that patients with tumor metastasis have decreased total T cells and CD4^+^ T cells in peripheral blood.^31^ Some researchers also observed the important role of peripheral blood CD4^+^ T cells in predicting the OS and chemotherapy effect of patients with pancreatic cancer, and they hypothesize that the CD4^+^ T cell compartment may have a critical role in response to chemoimmunotherapy treatment in metastatic pancreatic ductal adenocarcinoma.^13^

The thermal effect (ablation and hyperthermia), mechanical effect, and cavitation effect of focused ultrasound can induce tumor immune response by releasing tumor‐related antigens.^37^, ^38^, ^39^ Thermal ablation could increase the abundance of tumor antigens and be recognized by antigen‐presenting cells, thereby activating lymphocytes for a specific immune response.^39^ HIFU‐mediated mild‐moderate hyperthermia has been shown to increase the vascular and cellular permeability and enhance the metabolic activity of hyperthermia targets, which can enhance delivery/passage of immune cells. Although mild hyperthermia usually does not lead to immediate cell death, it can directly promote antigen cross‐presentation and the generation and expansion of tumor‐specific T cells in a variety of tumor settings.^39^ Mechanical destruction of the dense connective tissue in pancreatic cancer under the action of HIFU may create an improved microenvironment and increase the infiltration of immune cells near tumor cells.^40^ Mouratidis et al. treated murine orthotopic pancreatic tumor with HIFU, and reported an increase in CD8^+^ T cells in the blood after 6 days of treatment,^41^ which is similar to the results of this study. However, the above‐mentioned effects can occur simultaneously during the treatment period. Since, the study subjects were patients with unresectable pancreatic cancer, pathological specimens were not available for them, so it is not possible to clearly distinguish which effects occurred.

As we have mentioned, peripheral blood lymphocyte subsets were collected before and immediately after a session of treatment. Thus, the corresponding changes in peripheral blood lymphocyte subsets were closely related to the role of HIFU. The changes of peripheral blood lymphocyte subsets after HIFU treatment may reflect the overall immune status of patients, which may be the reason why the change of CD4^+^ T cell proportion was related to the patient's OS.

CA19‐9 is the best validated biomarker in pancreatic cancer, which can be used to evaluate the prognosis and therapeutic effect, and the normal baseline level of CA19‐9 was associated with long‐term survival.^42^ However, CA19‐9 in this group plays a limited role in predicting survival time. Although this group of patients was in the terminal stage, there are still 3/30 patients with CA19‐9 <5 U/mL and 6/30 patients' CA19‐9 <10 U/mL, so this part of patients cannot be excluded from Lewis antigen negative one. In addition, studies have shown that patients with negative Lewis antigen have a worse prognosis.^43^ This may be the reason that CA19‐9 cannot be used as an ideal biomarker to predict the prognosis of patients in this study.

Several limitations of the study should be acknowledged. First, all the patients received HIFU treatment at a single center. Second, the number of patients enrolled in this study was relative small, so our results should be confirmed by studies including larger sample sizes in the future. In addition, this study only analyzes the survival of parameters that are easy to obtain in clinical work after HIFU treatment, and did not conduct experimental research on the relevant mechanism, which still needs follow‐up research.

## 5 CONCLUSION

HIFU can effectively relieve pain in patients with unresectable pancreatic cancer. Higher VAS score and decreased CD4^+^ T cell ratio after HIFU treatment may be potential survival risk factors for patients with unresectable pancreatic cancer, which can be easily obtained in clinical work and help to evaluate patients' feedback on HIFU therapy early. We hope that these findings will provide new ideas for further application of HIFU treatment in patients with unresectable pancreatic cancer.

## AUTHOR CONTRIBUTIONS

**Yu Yang:** Conceptualization (equal); data curation (lead); investigation (lead); methodology (equal); writing – original draft (lead). **Xian‐Quan Shi:** Data curation (supporting); investigation (supporting); resources (equal); writing – review and editing (equal). **Guang Chen:** Investigation (supporting); methodology (supporting); resources (supporting). **linxue Qian:** Conceptualization (equal); funding acquisition (lead); methodology (equal); resources (equal); supervision (lead); writing – review and editing (lead).

## FUNDING INFORMATION

This work was supported by the General Program of National Natural Science Foundation of China (grant number: 52273300).

## CONFLICT OF INTEREST STATEMENT

The author reports no conflicts of interest in this work.

## Data Availability

The data that support the findings of this study are available on request from the corresponding author. The data are not publicly available due to privacy or ethical restrictions.
